# Multiple-Actuator Fault Isolation Using a Minimal *ℓ*_1_-Norm Solution with Applications in Overactuated Electric Vehicles

**DOI:** 10.3390/s22062144

**Published:** 2022-03-10

**Authors:** Jinseong Park, Youngjin Park

**Affiliations:** 1 Department of Artificial Intelligence Machinery, Korea Institute of Machinery & Materials, Daejeon 34103, Korea; 2 Department of Mechanical Engineering, Korea Advanced Institute of Science and Technology, Daejeon 34141, Korea; yjpark@kaist.ac.kr

**Keywords:** electric vehicle, minimal *ℓ*_1_-norm solution, multiple fault isolation, overactuated system

## Abstract

A multiple-actuator fault isolation approach for overactuated electric vehicles (EVs) is designed with a minimal $\ell_{1}$-norm solution. As the numbers of driving motors and steering actuators increase beyond the number of controlled variables, an EV becomes an overactuated system, which exhibits actuator redundancy and enables the possibility of fault-tolerant control (FTC). On the other hand, an increase in the number of actuators also increases the possibility of simultaneously occurring multiple faults. To ensure EV reliability while driving, exact and fast fault isolation is required; however, the existing fault isolation methods demand high computational power or complicated procedures because the overactuated systems have many actuators, and the number of simultaneous fault occurrences is increased. The method proposed in this paper exploits the concept of sparsity. The underdetermined linear system is defined from the parity equation, and fault isolation is achieved by obtaining the sparsest nonzero component of the residuals from the minimal $\ell_{1}$-norm solution. Therefore, the locations of the faults can be obtained in a sequence, and only a consistently low computational load is required regardless of the isolated number of faults. The experimental results obtained with a scaled-down overactuated EV support the effectiveness of the proposed method, and a quantitative index of the sparsity condition for the target EV is discussed with a CarSim-connected MATLAB/Simulink simulation.

## 1. Introduction

In recent years, many automatic control systems have employed redundant actuators to enhance their existing control strategies and robustness. These systems are defined as overactuated systems if the number of inputs is greater than the number of state variables. In-wheel motors (IWMs) have been applied to electric vehicles (EVs) to evaluate two- or four-wheel independent drive (4WID); these are useful in all conditions from the standpoints of driving, turning, stopping, and ride comfort [1,2]. For additional actuator subsystems, however, the possibility of fault occurrence is also increased. Thus, advanced fault-tolerant control (FTC) and fault diagnosis methods should be developed.

For safety-critical systems, FTC and fault detection and isolation (FDI) methods are needed to guarantee a redundant degree of system reliability. Most FTC studies have used the characteristics of actuator redundancy. An optimal reconfigurable control strategy has been developed by considering actuator redundancy in the presence of faults based on reliability indicators [3]. A computationally efficient distribution approach for control effort has also been proposed; it employs a weighted pseudoinverse-based control allocation (WPCA) method [4]. A sliding mode has also been used with control allocation for FTC [5].

To efficiently react to the faults, FDI techniques have been developed based on model-based approaches, such as parity relations, which is also called analytical redundancy relations (ARRs), unknown input observers [6], and adaptive techniques [7,8,9]. ARR-based FDI is usually more cost-effective but should be robust. Therefore, many robust FDI methods that generate residuals to maximize fault sensitivity and minimize sensitivity to other unexpected effects, such as noise, unknown disturbances, and model uncertainties, have been studied [10,11,12]. Structured residual analysis was introduced by applying transforming vectors to the parity equation to enhance the residuals so that each residual is completely affected by only one of the faults [13]. Using ARRs, the fault signature matrix (FSM) which attributes fault signatures for each fault is also adopted for fault isolation. The extended FSM was developed to improve fault isolability under multiple types of fault conditions by increasing the number of ARRs with additional dependent ARRs [14] or artificial output signal augmentation [15]. Sensitivity signature was studied to represent the consistency of ARRs by three terms for more distinguished signature generation and improved fault isolation abilities [16].

On the other hand, data-driven approaches for FDI also have been studied with the remarkable growth of artificial intelligence (AI) but applications are mainly biased towards rotating machinery [17,18]. Nevertheless, AI-based FDI for nonrotating systems was also conducted. The economical fault diagnosis by combining a sparse autoencoder and an echo state network was proposed to overcome low-quality attitude sensor measurements [19]. Deep autoencoder based anomaly detection and disambiguation of aircraft was proposed with multiple sensor data [20].

However, the aforementioned research mainly considers robust fault isolation for a single fault, not how the procedure becomes much more complicated when the number of simultaneously occurred faults increases. An increase in the number of faults requires more computational power and more complicated procedures for existing FDI methods. The transfer matrix from the faults to the residuals has been derived in terms of the eigenvalues of the fault detection filter with regard to the reconstruction of multiple simultaneous faults [21]; however, the characteristics of a system with redundant actuators are not considered. For overactuated systems, a geometric FDI approach has been extended with the development of sufficient conditions for the detection and isolation of simultaneous faults [22]. The residual generation procedure becomes more complicated as the number of actuators increases. Therefore, advanced actuator fault isolation methods that fully utilize the characteristics of overactuated systems are needed.

In this paper, model-based multiple-actuator fault isolation is considered for overactuated systems with the concept of sparsity. A sparse representation is one in which a small number of coefficients contain a large proportion of the total energy [23]. The minimal $\ell_{1}$-norm problem, which is a relaxed version of a minimal zero-norm (cardinality norm) problem, can be applied to obtain sparse solutions, while the $\ell_{2}$-norm minimization problems represent the scenario in which all of the coefficients contain similar energies. The concept of sparsity has been widely used in fault diagnosis because it possesses many practical benefits, such as its allowing us to make fewer measurements or store less data [24,25]. A fault detection strategy was proposed by using principal component analysis to achieve sparsity with the $\ell_{1}$-norm constraint [26]. Multivariate fault isolation was performed according to a variable selection problem with an $\ell_{1}$-norm constraint that was utilized as a surrogate for the $\ell_{0}$-norm. These methods were verified with a benchmark Tennessee Eastman process but not for a dynamic system such as an EV. Robust fault detection and isolation schemes with robust $\ell_{1}$ state estimators were proposed to develop appropriate threshold logic and significantly reduced false alarm rates were reported [27,28]. However, multiple faults were examined for a normal multi-input multi-output (MIMO) system, not for an overactuated system. If a system is overactuated, the sparse fault isolation solution from the residual equations, which can represent the number and locations of multiple faults, can be obtained by utilizing the $\ell_{1}$-norm minimization approach. A fault isolation approach similar to that developed in this paper was proposed for an underdetermined system based on a sparse estimation problem with the $\ell_{1}$-norm, but only a turbofan simulation model was applied [29]. The main contributions of this work are threefold, which can be summarized as follows.

1. Simultaneously occurring multiple-actuator faults are considered for FDI with fully utilizing the characteristics of overactuated system.
2. With representing the residual equation of the overactuated system as an underdetermined linear system, fault isolation can be achieved by obtaining the sparsest nonzero component of the residuals from the minimal $\ell_{1}$-norm solution. The computational load is consistently low regardless of the isolated number of faults.
3. The experiments with a scaled-down overactuated EV are performed to support the effectiveness of the proposed method. In addition, because the sparsity condition highly depends on the system characteristics, a quantitative analysis of sparsity for the target EV is discussed.

The rest of this paper is organized as follows. In Section 2, a summary of structural residual analysis with parity relations is presented. A multiple-fault isolation approach with the minimal $\ell_{1}$-norm solution based on parity relations is presented in Section 3. An application of our fault isolation method with an overactuated EV is presented in Section 4, and conclusive remarks are presented in Section 5.

## 2. Brief Summary of Structural Residual Analysis with Parity Relations

The proposed method utilizes a parity equation. Therefore, the basic concept of the input–output parity relation approach is briefly described.

### 2.1. Fault Model

The state-space equation for a linear system is represented as follows:

(1) $$\begin{array}{c} \begin{array}{c} \dot{x}\left(t\right)=Ax\left(t\right)+Bu\left(t\right) \end{array} \end{array}$$

(2) $$\begin{array}{c} \begin{array}{c} y(t)=Cx(t)+Du(t) \end{array} \end{array}$$

where $x\in R^{n\times 1}$ is the state variable; $u\in R^{m\times 1}$ is the control input; $y\in R^{p\times 1}$ is the measurement output, and $A\in R^{n\times n}$, $B\in R^{n\times m}$, $C\in R^{p\times n}$, and $D\in R^{p\times m}$.

Due to abnormal operations or material aging, faults may occur. In addition, as the number of actuators increases, the fault occurrence probability also increases. Actuator faults are generated as various types, such as additive (e.g., bias faults) and multiplicative (e.g., the loss of actuator effectiveness) faults [30,31,32,33]. In this paper, actuator faults are considered combinations of additive and multiplicative faults. The equation of the fault model can be represented based on the nominal system in Equations (1) and (2) as follows:

(3) $$\begin{array}{c} \begin{array}{c} \dot{x}\left(t\right)=Ax\left(t\right)+B\left[\left(I+\alpha \right)u\left(t\right)+f_{u}\right]\\ y\left(t\right)=Ax\left(t\right)+D[\left(I+\alpha \right)u\left(t\right)+f_{u}] \end{array} \end{array}$$

where $\alpha \in R^{m\times m}$ represents a multiplicative actuator fault and is a diagonal matrix with the diagonal elements $\alpha_{ii},i=1,\ldots ,m$ s.t. −1<α<0. An additive actuator fault is represented by $f_{u}\in R^{m\times 1}$. Both fault types are assumed to be time-invariant.

### 2.2. Multiple Fault Isolation with Structural Residual Analysis

The response of the fault model can be represented in the s-domain based on Equation (3) as follows:

(4) $$\begin{array}{c} \begin{array}{c} Y_{sensor}\left(s\right)=G\left(s\right)\left[\left(I+\alpha \right)U\left(s\right)+f_{u}\right] \end{array} \end{array}$$

where $G\left(s\right)=C{\left(sI-A\right)}^{-1}B+D$. For fault isolation in a dynamic system, residuals can be used to check the actual system behavior in terms of its consistency with the mathematical model. Residuals are generated from the difference between the measured output $Y_{sensor}\left(s\right)$ and the estimated output $Y_{model}\left(s\right)$, and $Y_{model}\left(s\right)$ is constructed from the fault-free reference model:

(5) $$\begin{array}{c} \begin{array}{c} R\left(s\right)=Y_{sensor}\left(s\right)-Y_{model}\left(s\right)\\ \;\;\;\;=G\left(s\right)\left[\left(I+\alpha \right)U\left(s\right)+f_{u}\right]-\left[G\left(s\right)U\left(s\right)\right]\\ \;\;\;\;=G_{1}\left(s\right)\left[\alpha_{1}U_{1}\left(s\right)+f_{u,1}\right]+\cdots +G_{m}\left(s\right)\left[\alpha_{m}U_{m}\left(s\right)+f_{u,m}\right] \end{array} \end{array}$$

where $R\left(s\right)\in R^{p\times 1}$.

The conventional structural residual method enhances the residuals by introducing transformation vectors so that only a fault-specific subset of the components is nonzero in response to a particular fault; this is formulated as follows [13]:

(6) $$\begin{array}{c} \begin{array}{c} R_{i}^{*}\left(s\right)=W_{i}^{T}R\left(s\right) \end{array} \end{array}$$

where $R_{i}^{*}\left(s\right)$ is the structural residual; $W_{i}^{T}$ is a transformation vector satisfying $W_{i}^{T}G_{i}=0$, $W_{i}^{T}G_{j}\neq 0$, and for i,j=1,…,m,j≠i.

With the structural residual sets $R_{i}^{*}\left(s\right)$, up to p−1 faults can be isolated because the transformation vectors $W_{i}^{T}$ can satisfy up to p−1 constraints. For a single-actuator fault isolation task, *m* $R_{i}^{*}\left(s\right)$ obtained by implementing *m* $W_{i}^{T}$s, which should be orthogonal to $G_{i}\left(s\right)$, are required so that we can investigate the unaffected signal(s) $R_{i}^{*}\left(s\right)$. The $C_{i}^{m}$ orthogonal $R_{i}^{*}\left(s\right)$ are needed for the case involving a single fault. However, as the number of faults increases by *k*, $C_{1}^{m}+C_{2}^{m}+\cdots +C_{k}^{m}$ structural residuals are needed. Furthermore, the transformation vector sets for each fault should be independent of the other fault cases, which is very hard to achieve. Additionally, because the number of faults that have occurred is unknown, sequential investigation is required, as explained in Algorithm 1. Consequently, the increases in the numbers of actuators (*m*) and fault occurrences (*k*) require many more combinations of structural residuals, and the iterative search procedure makes it difficult to isolate faults.
**Algorithm 1** Fault isolation with structural residual analysis.Input: *R*(s)1:
 *k*: the assumed number of simultaneously occurring faults
2:
 *is_ fault* ← *False*
3:
 **if**
$r_{i}\left(t\right)$ > *threshold* **then**
4:
    *is_ fault* ← *true*
5:
 **if**
*is_ fault*
**then**
6:
   **for** k≤m **do**
7:
    **for** $i\leq C_{k}^{m}$ **do**
8:
      Implement $C_{k}^{m}$$W^{T}$s
9:
      $R_{i}^{*}\left(s\right)\leftarrow W_{i}^{T}R\left(s\right)$
10:
    **if** $R_{i}^{*}\left(s\right)$ is isolated successfully **then**
11:
      break


## 3. Multiple Fault Isolation for an Overactuated System with a Minimal $\ell_{1}$-Norm Solution

The linear system $A_{sys}x_{sys}\left(t\right)=b_{sys}\left(t\right)$, where $A_{sys}\in R^{p\times m}$, $x_{sys}\in R^{m\times 1}$, $b_{sys}\in R^{p\times 1}$, is underdetermined when the number of unknowns *m* is larger than the number of equations *p*, p<m. An underdetermined system has an infinite number of solutions due to its redundant degrees of freedom. Among these solutions, if the number of equations *p* is sufficiently larger than the number of nonzero components in the solution x(t), the solution can be called sparse. The quantitative index of the sparsity bound is discussed in Section 4.3.

### 3.1. Residual Equation for Overactuated Systems

An overactuated system is equivalent to an underdetermined system. The input matrix B(t) in Equation (1) has more columns than rows. Therefore, B(t) is rank deficient, RANK(B)=s<m and *B* has a null space with m−s dimensions. In the same manner, G(S) in Equation (4) is also rank deficient.

As the number and locations of faults are investigated in the fault isolation step, Equation (5) can be simplified as follows:

(7) $$\begin{array}{c} \begin{array}{c} R\left(s\right)=G\left(s\right)[\alpha U\left(s\right)+f_{u}]=G\left(s\right)E\left(s\right) \end{array} \end{array}$$

When faults are detected, it is natural that the system does not operate dynamically until the system is put into the steady state by a feedback controller, such as electronic stability control (ESC), even if the behavior has a bias due to the presence of the faults. Therefore, without loss of generality, fault isolation is carried out in the steady state. Consequently, the system transfer function can be considered constant during the fault isolation step.

The residual with a constant transfer function is represented as follows:

(8) $$\begin{array}{c} \begin{array}{c} R(s)=GE(s) \end{array} \end{array}$$

where $G\in R^{p\times m}$ represents the zero-frequency response. The residual can be represented in the time domain as follows:

(9) $$\begin{array}{c} \begin{array}{c} r(t)=Ge(t) \end{array} \end{array}$$

where r(t) and e(t) are the inverse Laplace transforms of R(s) and E(s), respectively. Therefore, the residual equations of the overactuated system can be represented as a linear system in the time domain.

### 3.2. $\ell_{1}$-Norm Minimization for the Sparsest Solution

Fault isolation can be accomplished through the linear system of Equation (9); the residuals r(t) do not need to be structured. To illustrate the concept of sparsity, the solution of $\ell_{1}$-norm minimization is compared with the minimal $\ell_{2}$-norm solution with an example, as follows: 

                         $G=\left[\begin{array}{ccccc} 1 & 0 & 0 & 0 & 1\\ 0 & 2 & 0 & 0 & 2\\ 0 & 0 & 3 & 0 & 3\\ 0 & 0 & 0 & 4 & 4 \end{array}\right],$ $r=\left[\begin{array}{c} 1\\ 0\\ 3\\ 0 \end{array}\right]$

The $\ell_{2}$-norm minimization is generally used to obtain minimum-energy solutions in which all components have similar magnitudes instead of some components having most of the energy.

(10) $$\begin{array}{c} \begin{array}{c} e_{2}\left(t\right)=\arg\min_{e}{∥\begin{array}{c} e(t) \end{array}∥}_{2}\\ \mathrm{subject}\;\mathrm{to}\;Ge(t)=r(t) \end{array} \end{array}$$

The solution of Equation (10) has a closed-form formulation, $e_{2}\left(t\right)=G^{+}r\left(t\right)$, where $G^{+}$ is the pseudoinverse of *G*, as represented in Appendix A. In the example, the minimal $\ell_{2}$-norm solution is $e_{2}={\left[\begin{array}{ccccc} 0.6 & 0.4 & 0.6 & 0.4 & 0.4 \end{array}\right]}^{T}$. All components of $e_{2}$ have similar magnitudes and are unrealistic from the perspective of fault isolation because they indicate that faults occur at all actuators. It is more natural for the residual to be perturbed by less (one or two) faults than for all the faults to occur with similar magnitudes. Therefore, the sparsest solution is more suitable for the fault isolation problem.

The general $L_{p}$-norm is defined mathematically as follows:

(11) $$\begin{array}{c} \begin{array}{c} {∥\begin{array}{c} e \end{array}∥}_{p}=\sqrt[{p}]{\sum_{i}e_{i}^{p}} \end{array} \end{array}$$

Such results hold for p>1. When p→0, the result is called (in a slight abuse of terminology) the minimization of the zero norm of *e* and is defined as follows [34,35]:

(12) $$\begin{array}{c} {∥e∥}_{0}^{0}=\mathrm{card}\left\{e_{i}|e_{i}\neq 0\right\} \end{array}$$

The minimal zero-norm solution obtains the nonzero components of e(t) with the minimum cardinality. Note that the cardinality denotes the number of elements in the set. Therefore, the sparsest solution can be obtained with Equation (12). In general, however, obtaining the sparsest solution for a general underdetermined system of equations is a nondeterministic polynomial-time (NP)-hard problem [36]. Solving the zero-norm minimization problem requires combinatorial optimization and is impractical. Instead, the $\ell_{1}$-norm is, in some sense, the convex relaxation of the zero norm. Furthermore, it has been proven that when the answer to the zero-norm minimization problem is sparse, it can be the same as the answer to $\ell_{1}$-norm minimization [24]. Therefore, $\ell_{1}$-norm optimization can be utilized to obtain the sparsest solutions that guarantee sparsity. The proposed multiple fault isolation method with the $\ell_{1}$-norm minimization approach is defined as follows:

(13) $$\begin{array}{c} \begin{array}{c} e_{1}\left(t\right)=\arg\min_{e}{∥\begin{array}{c} e(t) \end{array}∥}_{1}\\ \mathrm{subject}\;\mathrm{to}\;Ge(t)=r(t) \end{array} \end{array}$$

The number and locations of the nonzero components of the solution $e_{1}$(*t*) represent the number and locations of simultaneously occurring faults. The magnitude of each nonzero component indicates the fault energy corresponding to the actuators. In practice, residuals are used via averaging under steady-state conditions to eliminate sensor noise. The whole procedure of the proposed fault isolation method is represented in Algorithm 2. From Algorithm 2, it is evident that the solution can be obtained in a single sequence, contrary to that of structural residual analysis. The computational time of the $\ell_{1}$-norm-based fault isolation approach is influenced only by the size of the resultant transfer function *G* but is independent of the number of fault occurrences.
**Algorithm 2** Fault isolation with the minimal $\ell_{1}$-norm solution.Input: G(s), *r*(t)1:
 G←G(0)
2:
 w←The number of data points to be averaged
3:
 *is_ fault* ← *False*
4:
 **if**
$r_{i}\left(t\right)$ > *threshold* **then**
5:
    *is_ fault* ← *true*
6:
 **if**
*is_ fault* & *steady state* **then**
7:
    **for** k<w **do**
8:
    $r_{m}$ ← $r_{m}+r\left(k\right)$
9:
    $r_{m}\leftarrow r_{m}/w$
10:
    $e_{1}\left(0\right)\leftarrow G^{+}r_{m}$
11:
    $e_{1}$ ← *primal-dual algorithm*($e_{1}\left(0\right),G,r_{m}$)
12:
 **return**
*the number and locations of the nonzero components of*
$e_{1}$


No universally accepted definition of sparsity is available [23]. Many measures indicate the signal sparsity level of the system, but sparsity depends greatly on the system characteristics. Although closed-form solutions still cannot be obtained for $\ell_{1}$-norm minimization problems, many useful algorithms provide numerical solutions. In this paper, a primal-dual algorithm for linear programming is used to solve the $\ell_{1}$-norm minimization problem [37].

Considering the above example again, the $\ell_{1}$-optimization solution of Equation (13) is $e_{1}={\left[\begin{array}{ccccc} 1 & 0 & 1 & 0 & 0 \end{array}\right]}^{T}$. Only two nonzero components are contained in the solution, which is sparse compared to the $\ell_{2}$-norm minimization solution. Therefore, the minimal $\ell_{1}$-norm solution is more suitable for the concept of the fault isolation problem.

Although only the actuator fault isolation of the overactuated system is the focus in this paper, the sensor fault isolation also can be achieved. The detailed procedure is represented in Appendix B.

## 4. Application to an Overactuated EV

### 4.1. Dynamic Vehicle Model

A two-degree-of-freedom (DOF) bicycle model with a linear tire model is used as in [2] because the proposed fault isolation method is executed when the vehicle response is in a steady state or transient state with a small slip angle and slip ratio, guaranteeing vehicle stability even in the presence of the fault.

**Figure 1:**
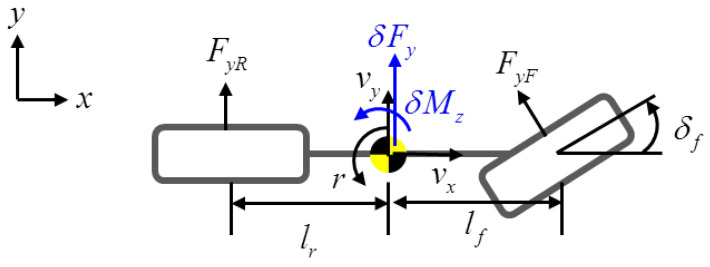
The 2-DOF bicycle model.

**Figure 2:**
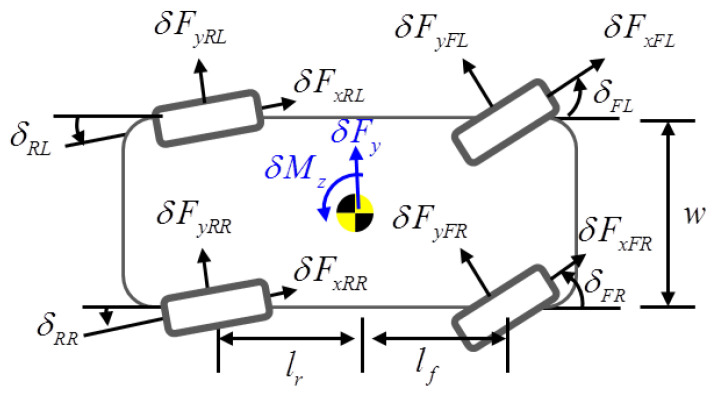
Tire forces generated from 4WD and 4WS actuators.

A schematic of the vehicle model is shown in Figure 1. The target vehicle has eight actuators that consist of four-wheel drive (4WD) and four-wheel steering (4WS) actuators, as represented in Figure 2. The state-space equation for the overactuated bicycle model is represented as follows:

(14) $$\begin{array}{c} \begin{array}{c} \dot{x}\left(t\right)=Ax\left(t\right)+Bu\left(t\right)+B_{f}\left(t\right)\delta_{f}\left(t\right) \end{array} \end{array}$$

(15) $$\begin{array}{c} \begin{array}{c} y(t)=Cx(t) \end{array} \end{array}$$

and

$$\begin{array}{c} x={\left[\begin{array}{cc} V_{y} & \gamma \end{array}\right]}^{T}\\ u={\left[\begin{array}{cccccccc} \delta F_{yFL} & \delta F_{yFR} & \delta F_{yRL} & \delta F_{yRR} & \delta F_{xFL} & \delta F_{xFR} & \delta F_{xRL} & \delta F_{xRR} \end{array}\right]}^{T}\\ A=\left[\begin{array}{cc} -\frac{2C_{f}+2C_{r}}{MV_{x}} & -V_{x}-\frac{l_{f}\cdot 2C_{f}-l_{r}\cdot 2C_{r}}{MV_{x}}\\ -\frac{2l_{f}\cdot C_{f}-2l_{r}\cdot C_{r}}{I_{z}V_{x}} & -\frac{l_{f}^{2}\cdot 2C_{f}+l_{r}^{2}\cdot C_{r}}{I_{z}V_{x}} \end{array}\right]\\ B=\left[\begin{array}{cccccccc} \frac{1}{M} & \frac{1}{M} & \frac{1}{M} & \frac{1}{M} & 0 & 0 & 0 & 0\\ \frac{l_{f}}{I_{z}} & \frac{l_{f}}{I_{z}} & -\frac{l_{r}}{I_{z}} & -\frac{l_{r}}{I_{z}} & -\frac{w}{2I_{z}} & \frac{w}{2I_{z}} & -\frac{w}{2I_{z}} & \frac{w}{2I_{z}} \end{array}\right]\\ B_{f}=\left[\begin{array}{cc} \frac{2C_{f}}{M} & \frac{2l_{f}C_{f}}{I_{z}} \end{array}\right]\\ T_{ij}=r_{w}\cdot \delta F_{xij},\;\Delta \delta_{ij}=\delta F_{yij}/C_{i} \end{array}$$

where n=2,m=8, $V_{y}$ is the lateral velocity; γ is the yaw rate; $\delta F_{xij}$ is longitudinal tire force; $\delta F_{yij}$ is lateral tire force; $C_{f}$ and $C_{r}$ are the cornering stiffness values of the front and rear wheels, respectively; *M* is the vehicle mass; $V_{x}$ is the longitudinal velocity; $l_{f}$ and $l_{r}$ are the distances from the center of gravity to the front and rear axles, respectively; $I_{z}$ is the yaw moment of inertia; *w* is the track width; $T_{ij}$ is the torque; $\delta_{ij}$ is the steering angle; $\delta_{f}$ is the feedforward steering angle commanded by the driver; $r_{w}$ is the radius of a wheel, and *C* is the identity matrix for full-state feedback (p=n).

### 4.2. Experimental Results

**Figure 3:**
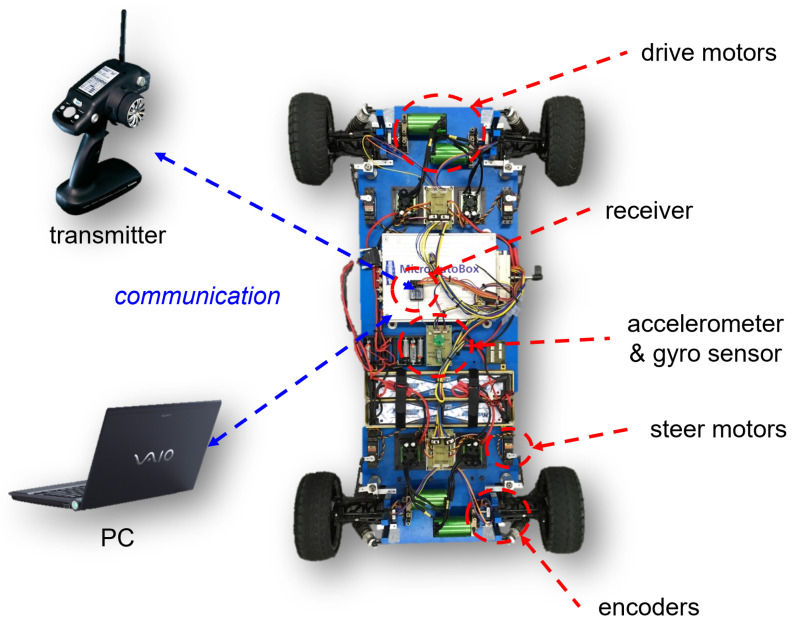
Scaled-down overactuated vehicle (1/5).

The proposed fault isolation method is experimentally verified in a real environment with the realization of an overactuated scaled-down EV, as illustrated in Figure 3. The scaled-down vehicle is approximately 1/5 the size of a small passenger vehicle. Each wheel is equipped with driving and steering motors, and a total of eight actuators are used. A 3-axis accelerometer, a yaw rate gyro sensor, and an encoder in each wheel are used to estimate the state variables of the bicycle model, such as the lateral velocity and yaw rate, as well as the longitudinal velocity.

The algorithms are implemented by using a MicroAutoBox electronic control unit (ECU) of dSPACE. A remote transmitter is used for driving and steering commands. A remote PC is also used for signal monitoring. The identification of the motor parameters and cornering stiffness values, and the use of observers to estimate the state variables from the sensor measurements were performed in our previous work [38]. The parameters of the vehicle are presented in Table 1.

**Figure 4:**
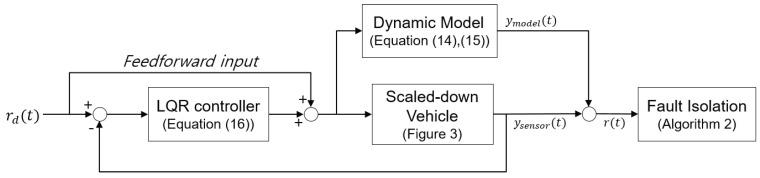
The schematic diagram of experiment with scaled-down vehicle.

The experiment is performed for a steady-state cornering maneuver with a feedforward front steering input of 10°. A linear quadratic controller is designed to obtain the gain *K* in Equation (16) with a closed-loop eigenvalue of $\lambda =-90\pm 90\sqrt{2}$i.

(16) $$\begin{array}{c} \begin{array}{c} u\left(t\right)=-K(y\left(t\right)-r_{d}\left(t\right)) \end{array} \end{array}$$

where $r_{d}$ is the desired state. The whole procedure of the experiment with a scaled-down vehicle is represented in Figure 4.

**Figure 5:**
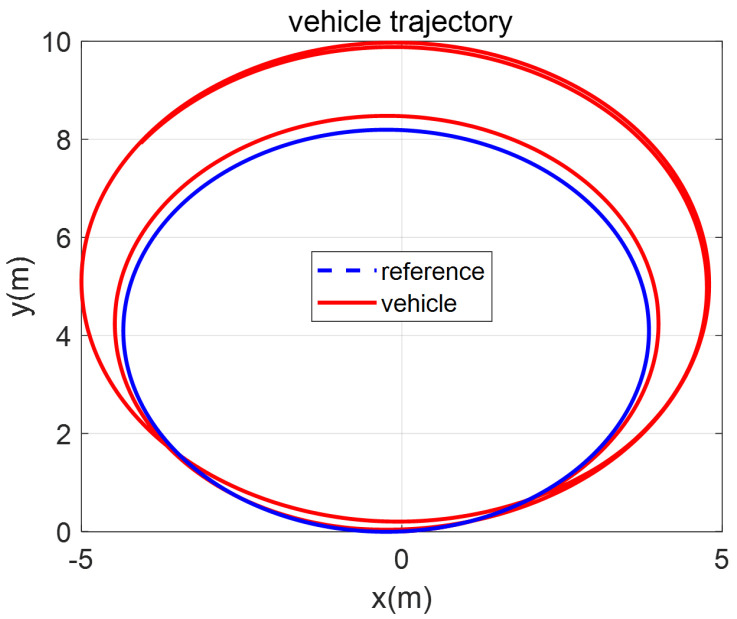
Vehicle trajectories in the steady-state cornering maneuver before and after fault occurrence.

**Figure 6:**
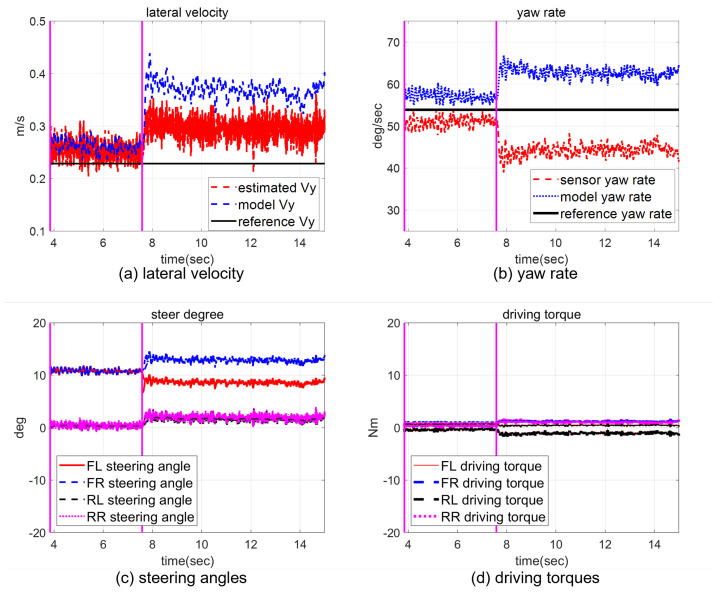
The closed-loop responses in the steady-state cornering maneuver. A fault occurs at 6.62 s.

In a scaled-down vehicle experiment, single fault isolation is performed to verify the feasibility of our method in a real environment (multiple fault generation makes it hard for our scaled-down vehicle to ensure safety), and the multiple faults are analyzed through a CarSim-based simulation in the next chapter. A fault is introduced in the front-left steering actuator $\delta_{fl}$ with a magnitude of $\alpha =-0.1,f_{u}=-3$. The vehicle trajectory for the whole experimental period and the response of the closed-loop system, including the fault occurrence, are represented in Figure 5 and Figure 6. After a fault occurs, the biases of the states $V_{r}$ and γ from the reference trajectory appear, as shown in Figure 6a,b. Although the controller tries to compensate for the fault, the effect of the fault remains. While the magnitude of the bias depends on the controller performance, a high control gain is not always a good choice in practice due to the presence of sensor noise. Nevertheless, the vehicle does not diverge from the target circle trajectory because of the controller, but the radius increases, as shown in Figure 5. In detail, the faulty steering actuator $\delta_{fl}$ cannot be exactly recovered to 10° by the controller, as shown in Figure 6c. At the same time, the rest of the actuators are also changed to create a counter yaw moment to compensate for the effect of the faulty actuator, as shown in Figure 6c,d.

A mathematical model ($Y_{model}\left(s\right)$ in Equation (5)) cannot exactly estimate $V_{y},\gamma$ after a fault occurs since no fault information is contained in the model. Generally, when a fault occurs, fault detection is performed based on the undesirable response, and then the fault is sequentially isolated and identified so that the fault can be compensated successfully by reconfiguration strategies such as FTC [3,39]. For conducting fault isolation with the proposed method, linearly independent columns of transfer function are needed. However, G(0) obtained from Equations (14) and (15) has only three linearly independent columns. Therefore, five more sensor measurements such as an acceleration sensors, $a_{x}\left(={\dot{V}}_{x}-{\dot{V}}_{y}\gamma \right)$, and four torque sensors, $u_{k}=T_{k}/r,\;k=2,4,5,6$, are augmented to a transfer function G(0) as follows:

(17) $$\begin{array}{c} \begin{array}{c} r\left(t\right)=G_{A}e\left(t\right) \end{array} \end{array}$$

where

$$\begin{array}{c} G_{A}={\left[\begin{array}{ccc} G_{acc} & G(0) & G_{u} \end{array}\right]}^{T}\\ G_{acc}=\left[\begin{array}{cccccccc} -\sin\delta_{fl}\left(t\right) & -\sin\delta_{fr}\left(t\right) & -\sin\delta_{rl}\left(t\right) & -\sin\delta_{rr}\left(t\right) & \;\cos\delta_{fl}\left(t\right) & \;\cos\delta_{fr}\left(t\right) & \;\cos\delta_{rl}\left(t\right) & \;\cos\delta_{rr}\left(t\right) \end{array}\right]\\ G_{u}=\left[\begin{array}{cccccccc} 0 & 1 & 0 & 0 & 0 & 0 & 0 & 0\\ 0 & 0 & 0 & 1 & 0 & 0 & 0 & 0\\ 0 & 0 & 0 & 0 & 1 & 0 & 0 & 0\\ 0 & 0 & 0 & 0 & 0 & 1 & 0 & 0 \end{array}\right]\\ r={\left[\begin{array}{ccccccc} a_{x} & V_{y} & \gamma & u_{2} & u_{4} & u_{5} & u_{6} \end{array}\right]}_{sensor}^{T}-{\left[\begin{array}{ccccccc} a_{x} & V_{y} & \gamma & u_{2} & u_{4} & u_{5} & u_{6} \end{array}\right]}_{model}^{T} \end{array}$$

**Figure 7:**
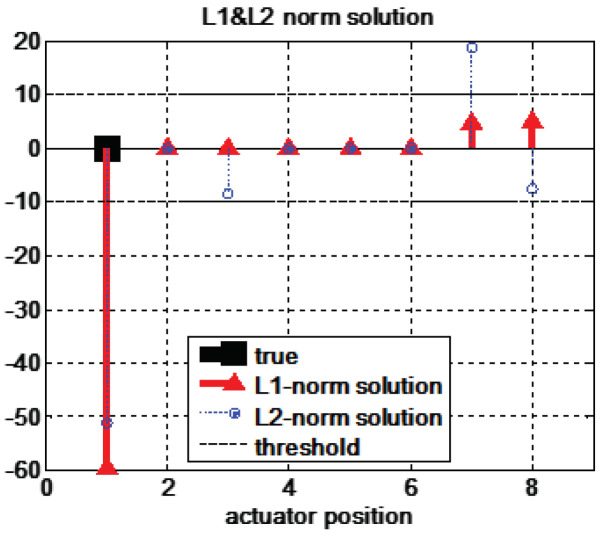
The experimental results of fault isolation with minimal $\ell_{1}$-norm and $\ell_{2}$-norm solution in a steady-state cornering maneuver. The actuator positions are 1(FL), 2(FR), 3(RL), and 4(RR) for the steering angles and 5(FL), 6(FR), 7(RL), and 8(RR) for the driving torques.

The *r* signal is obtained by averaging 1000 data points under the steady-state condition. The $\ell_{2}$-norm solution is adopted as the initial value of the primal-dual algorithm. The actuator fault isolation results are represented in Figure 7. The proposed method isolates a faulty actuator $\delta_{fr}$ with a remarkably large magnitude of fault energy. Although fault energy is also represented in other actuators, such as the rear-left and rear-right driving motors, due to the effect of sensor noise, their magnitudes are relatively small compared to the energy of a real faulty actuator. Additionally, the energies of the rear-left and rear-right driving actuators are identical, so they can be neglected by introducing the constraint in which faults rarely have the same energy, especially near the noise level. In the case of the $\ell_{2}$-norm-based method, a faulty actuator is also isolated with large fault energy. However, nonzero faulty energy occurs for three more actuators. Furthermore, the fault energy of the rear-left driving actuator seems to be higher than that of the other two actuators (the rear-left steering actuator and the rear-right driving actuator). In practice, the threshold for isolation is determined experimentally by considering the system characteristics or the level of sensor noise. However, it is possible to isolate the rear-left driving actuator as a faulty actuator with the minimal $\ell_{2}$-norm solution. Therefore, the experimental results support the notion that the proposed $\ell_{1}$-norm-based method is more accurate for fault isolation.

Structural residual analysis is also conducted for the comparison with our method. For a single fault isolation, *m* number of transforming vector $W^{T}$ should be implemented as follows:

(18) $$\begin{array}{c} W^{T}=\left[\begin{array}{ccccccc} 1000G_{A}\left(2,1\right)+G_{A}\left(3,1\right) & -1000G_{A}\left(1,1\right) & -G_{A}\left(1,1\right) & 1 & 1 & 1 & 1\\ 1000G_{A}\left(2,2\right)+G_{A}\left(3,2\right)+2G_{A}\left(4,2\right) & -1000G_{A}\left(1,2\right) & -G_{A}\left(1,2\right) & -2G_{A}\left(1,2\right) & 1 & 1 & 1\\ 1000G_{A}\left(2,3\right)+G_{A}\left(3,3\right) & -1000G_{A}\left(1,3\right) & -G_{A}\left(1,3\right) & 1 & 1 & 1 & 1\\ 1000G_{A}\left(2,4\right)+G_{A}\left(3,4\right)+2G_{A}\left(5,4\right) & -1000G_{A}\left(1,4\right) & -G_{A}\left(1,4\right) & 1 & -2G_{A}\left(1,4\right) & 1 & 1\\ 1000G_{A}\left(2,5\right)+G_{A}\left(3,5\right)+2G_{A}\left(6,5\right) & -1000G_{A}\left(1,5\right) & -G_{A}\left(1,5\right) & 1 & 1 & -2G_{A}\left(1,5\right) & 1\\ 1000G_{A}\left(2,6\right)+G_{A}\left(3,6\right)+2G_{A}\left(7,6\right) & -1000G_{A}\left(1,6\right) & -G_{A}\left(1,6\right) & 1 & 1 & 1 & -2G_{A}\left(1,5\right)\\ 1000G_{A}\left(2,7\right)+G_{A}\left(3,7\right) & -1000G_{A}\left(1,7\right) & -G_{A}\left(1,7\right) & 1 & 1 & 1 & 1 \end{array}\right] \end{array}$$

**Figure 8:**
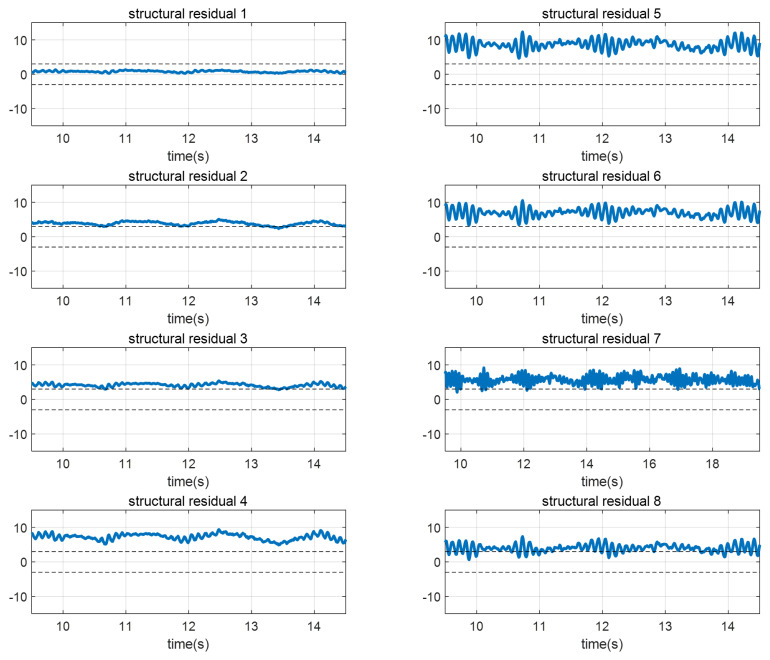
The experimental results of fault isolation with structural residual analysis in a steady-state cornering maneuver. The residuals indicate 1(FL), 2(FR), 3(RL), and 4(RR) for the steering actuator faults and 5(FL), 6(FR), 7(RL), and 8(RR) for the driving actuator faults.

The results represented that structural residual 1 indicating $\delta_{fl}$ fault is under the threshold while other residuals are over the threshold as shown in Figure 8. Therefore, the conventional method can isolate a single fault. The implementation of transforming vectors for a single fault is represented in Equation (18). However, for the multiple faults case, the successful implementation of $W^{T}$ is very hard to achieve because a much greater number of transforming vectors are needed and each row of $W^{T}$ should be designed to be consistent only for the particular combinations of multiple faults. Note that the required number of transforming vectors will be discussed subsequently.

On the other hand, the proposed method does not require residual to be structured for the consistency of a particular combination of faults. If the columns of the transfer function of the system model are linearly independent and the sparsity is satisfied, FDI can be achieved with a single sequence of linear programming procedures regardless of the occurred number of faults. In summary, our method is obviously more accurate than the $\ell_{2}$-norm-based method, and more simple than the conventional structural method.

### 4.3. Discussion

The solution of our method, Equation (13), is sparse if the number of outputs *p* is sufficiently larger than the number of faults (the nonzero component of e(t)). Therefore, a minimally required number of outputs should be investigated for multiple fault isolation to guarantee sparsity. First, in this section, we discuss the multiple fault isolation performance of our method with the maximum allowable number of outputs (p=m−1) while maintaining an underdetermined system as represented in Equation (17). It is worth mentioning that if p≥m, the system becomes overdetermined, and fault isolation can be achieved directly; this condition is not a focus of this paper. Second, the condition of the sparsity bound of our overactuated EV, which is a quantitative index of the aforementioned expression regarding a “sufficiently large number of outputs”, is derived.

Simulations are performed with the vehicle simulation package CarSim, which is connected to MATLAB/Simulink. The simulation conditions are that the sampling rate is dt=0.001 s and that the sensor is contaminated by zero-mean white noise with a covariance of $S=[2\times 10^{-6}\;0;\;0\;4\times 10^{-6}]$. The vehicle speed is 50 km/h, and the feedforward driver input contains 2° angles for each front steering actuator for steady-state cornering. For each case, all of the introduced faults have magnitudes of α=−0.5 and $f_{u}=-0.5$. After faults occur, a conventional linear quadratic controller tries to stabilize the system. Note that the threshold for detecting faults is set to 500 for $e_{A}\left(t\right)$ in this simulation.

**Figure 9:**
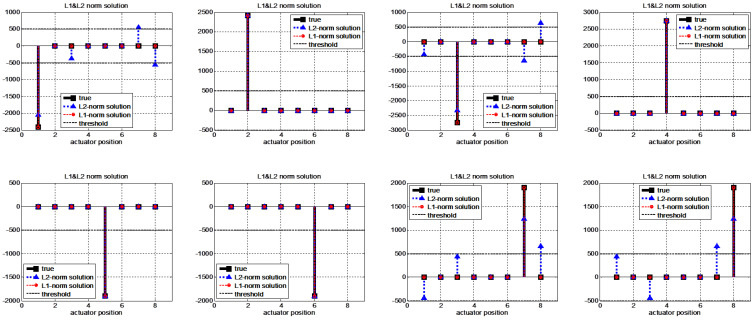
Single fault isolation results for different locations with the $\ell_{1}$-norm and $\ell_{2}$-norm minimization methods. The true signal indicates the true magnitude of e(t), and the minimal $\ell_{1}$-norm and $\ell_{2}$-norm solution signals indicate the estimated magnitude of e(t).

The fault isolation performance of the minimal $\ell_{1}$-norm solution is compared with that of the minimal $\ell_{2}$-norm solution. The structured residual method is compared in terms of the computational load rather than the accuracy of the isolation results because implementing independent transformation vectors is a much more difficult procedure for multiple fault cases. The single-actuator fault isolation results are represented in Figure 9. The minimal $\ell_{1}$-norm solutions exactly isolate the faults for all actuator fault cases. On the other hand, the minimal $\ell_{2}$-norm solutions obtain accurate fault positions but also indicate incorrect fault quantities and positions in four cases (cases 1, 3, 7, and 8). These results support the accuracy of our method; the minimal $\ell_{1}$-norm solution represents that the fault energy is concentrated in the sparsest number of faults, while the minimal $\ell_{2}$-norm solution represents that the fault energy is distributed to many actuators. If the conventional structural residual method is applied, eight independent transformation vectors are required for single-actuator fault isolation.

**Figure 10:**
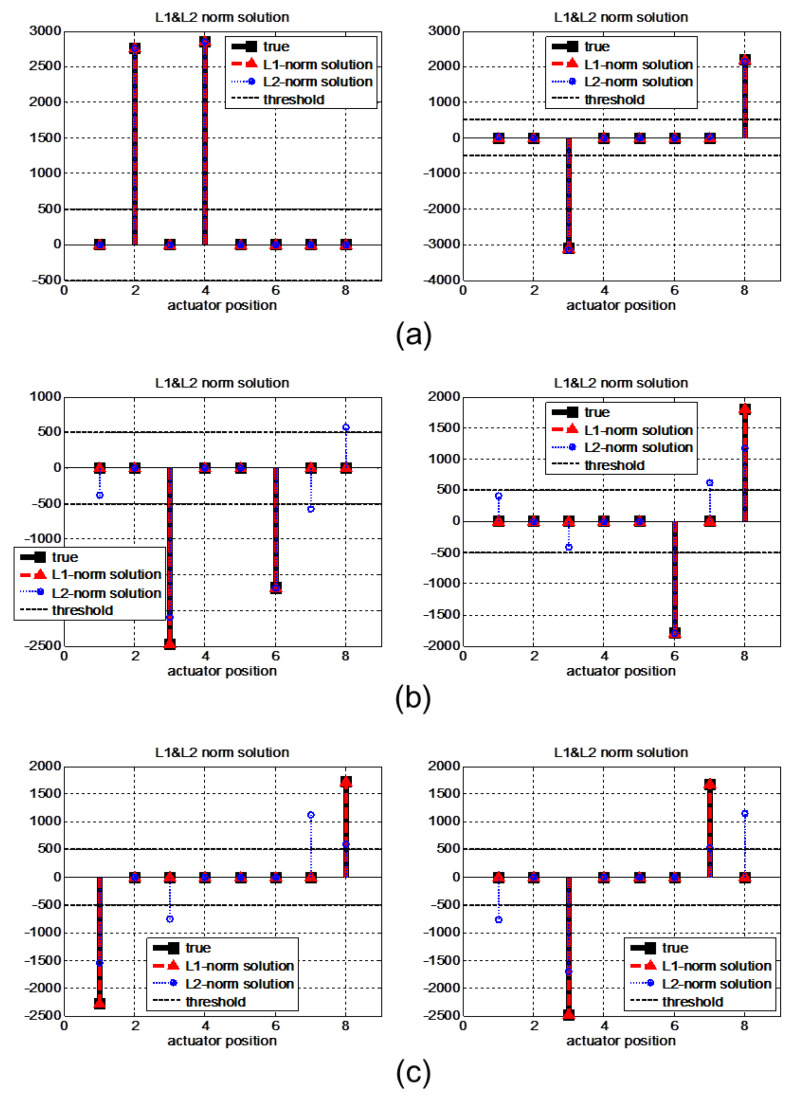
Two simultaneous fault isolation results obtained with the $\ell_{1}$-norm and $\ell_{2}$-norm minimization methods. The minimal $\ell_{1}$-norm solutions isolate the faults exactly for all represented cases, but minimal $\ell_{2}$-norm solutions represent (**a**) correct, (**b**) false alarm, and (**c**) false alarm & missed detection cases.

When faults occur in two actuators simultaneously, the possible number of cases is $C_{2}^{8}=28$. The isolation results obtained for several cases are represented in Figure 10. With the proposed method, the two actuator faults in each of the represented cases can be exactly isolated. On the other hand, the results of the $\ell_{2}$-norm method are worse than the results of the single fault case; most of the results contain more than one isolated actuator. In this case, if structural residual analysis is applied, $C_{1}^{8}+C_{2}^{8}=8+28=36$ independent transformation vectors are needed.

**Figure 11:**
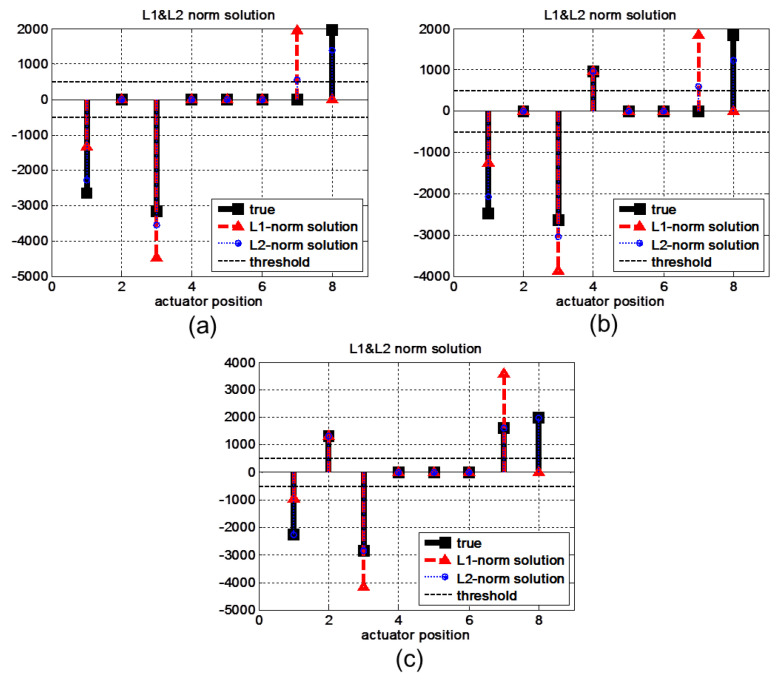
Isolation results obtained by the $\ell_{1}$-norm and $\ell_{2}$-norm minimization methods in cases with three or more simultaneous faults. Because sparsity cannot be guaranteed, the $\ell_{1}$-norm solutions cannot exactly isolate faults. (**a**) Three, (**b**) four, and (**c**) five faults.

When faults occur simultaneously at more than two actuators, even $\ell_{1}$-norm minimization cannot isolate the faults exactly because the sparsity is no longer guaranteed for this target system, as represented in Figure 11. The results include not only exact locations of faults but also incorrect locations that cause false alarm and missed detection problems. The results of the proposed method become similar to those of the $\ell_{1}$-norm method as the number of faults increases. The reason for this is that the sparsity of the isolation problem is no longer guaranteed for more than two faults for a given number of outputs *p*. Therefore, the $\ell_{1}$-norm problem represents the trend of the minimal energy solution, similar to the $\ell_{1}$-norm solution.

**Figure 12:**
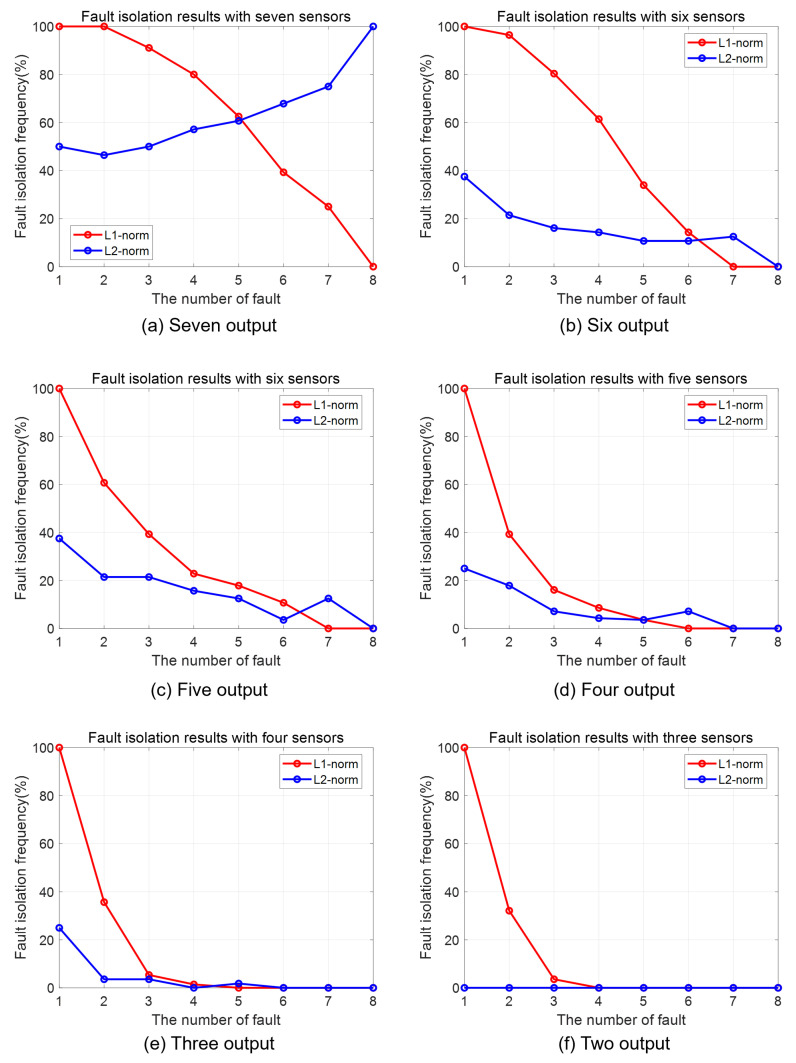
The exact fault isolation rates of the two compares methods for different numbers of outputs and faults. (**a**) $V_{y},\gamma ,a_{x},u_{2},u_{4},u_{5},u_{6}$, (**b**) $V_{y},\gamma ,a_{x},u_{2},u_{4},u_{5}$, (**c**) $V_{y},\gamma ,a_{x},u_{2},u_{4}$, (**d**) $V_{y},\gamma ,a_{x},u_{2}$, (**e**) $V_{y},\gamma ,a_{x}$, and (**f**) $V_{y},\gamma$ output cases.

The sparsity of our target system can be determined experimentally with the results represented in Figure 12. The exact fault isolation rates of the two methods are shown for the numbers of outputs and faults. When seven outputs are used, as shown in Equation (17), the fault isolation results are represented in Figure 12a; the isolation performance with $\ell_{1}$-norm minimization is perfect for up to two simultaneous fault occurrences, and the accuracy decreases monotonically as the number of faults that occur increases. On the other hand, the $\ell_{2}$-norm minimization solution is perfect only when all of the actuators have faults because the situation suits the concept of a minimal-energy solution (the fault energy is evenly distributed), but such a case is unrealistic. Therefore, Figure 12a exactly reflects the aforementioned simulation results. The multiple fault isolation results obtained according to a decreasing number of outputs are represented in Figure 12b–f; in each case, the configurations of the outputs are (b) $V_{y},\gamma ,a_{x},u_{2},u_{4},u_{5}$, (c) $V_{y},\gamma ,a_{x},u_{2},u_{4}$, (d) $V_{y},\gamma ,a_{x},u_{2}$, (e) $V_{y},\gamma ,a_{x}$, and (f) $V_{y},\gamma$. As the number of outputs decreases, the fault isolation performance also decreases for both methods. If the number of outputs is six, as represented in Figure 12b, $\ell_{1}$-norm method can perfectly isolate the fault for the one-fault case and can isolate with 96.5% effectiveness for the two-fault case. When the number of sensors is less than five, only one fault can be exactly isolated. From the trends shown in Figure 12, the sparsity bound of this system can be inferred experimentally as floor(p/3).

Our method requires only O(p) iterations for linear programming regardless of the number of fault occurrences. On the other hand, structural residual analysis isolates faults sequentially until the structural residuals are successfully isolated; therefore, $C_{1}^{8}+C_{2}^{8}+\cdots +C_{k}^{8}$ calculations are needed to obtain linearly independent transformation vectors. In fact, the implementation of independent transformation vectors is a very difficult process.

## 5. Conclusions

A multiple-actuator fault isolation method for overactuated EVs is proposed based on the concept of sparsity. The $\ell_{1}$-norm minimization problem can be adopted to obtain the sparsest solution. The system is underdetermined when the number of inputs is larger than the number of state variables. Sparsity can be guaranteed with mathematical models that possess redundant DOFs so that the number of output variables is sufficiently larger than the number of simultaneously occurring faults. The location and quantity of multiple faults can be isolated in one sequence within O(p) iterations; this approach is much more efficient than the conventional structural residual analysis. The experimental results obtained with a 1/5 scaled-down overactuated EV support the effectiveness of the proposed method; the minimal $\ell_{1}$-norm solution is accurate for fault isolation, while the $\ell_{2}$-norm minimization solution includes both correct and incorrect fault positions. To verify the sparsity bound of our target system, fault isolation under various numbers of outputs and faults is performed with a CarSim model that is connected to MATLAB/Simulink. To guarantee the sparsity of the target overactuated EV for fault isolation, it is verified that the number of outputs should be three times greater than the number of simultaneous faults. Therefore, our proposed fault isolation method is more accurate than the $\ell_{2}$-norm and more efficient than structural residual-based methods for overactuated EVs with up to two simultaneous fault occurrences, which is a sufficiently practical condition in reality.

Our future research directions will be achieving fault isolation in situations with unexpected effects such as external disturbances or model uncertainty. Additional observers such as an adaptive parameter observer or a disturbance observer can be integrated into the fault isolation method to enhance isolation performance. Extension of our method to the parameter fault isolation also can be a possible direction for future work. The proposed method has difficulties achieving parameter fault isolation when the number of parameter types is over the maximum isolable number of fault type *m* (the number of inputs). In addition, parameter faults are hard to distinguish from the actuator faults within a linear system structure. The augmentation of additional residuals consistent with each particular parameter or fusion with parameter identification approaches can be a possible solution.

## Figures and Tables

**Table 1:** Parameters of the scaled-down vehicle.

Parameters	Values
m	14.75 kg
$l_{f}$	0.374 m
$l_{r}$	0.366 m
*w*	0.48 m
$r_{w}$	0.08 m
$I_{z}$	1.077 m
$C_{f}$	555 Ns/rad
$C_{r}$	450 Ns/rad

## Data Availability

Not applicable.

## Appendix A. The Minimal *ℓ*_2_ -Norm Solution

To obtain the solution of Equation (10), a Lagrange multiplier is introduced:

(A1) $$\begin{array}{c} \begin{array}{c} L\left(e\left(t\right),\lambda \right)=e^{T}\left(t\right)e\left(t\right)+\lambda \left(Ge\left(t\right)-r\left(t\right)\right) \end{array} \end{array}$$

The necessary conditions for optimality are as follows:

(A2) $$\begin{array}{c} \begin{array}{c} {▽}_{e}L=2e^{T}\left(t\right)+\lambda G=0 \end{array} \end{array}$$

(A3) $$\begin{array}{c} \begin{array}{c} {▽}_{\lambda }L=Ge\left(t\right)-r\left(t\right)=0 \end{array} \end{array}$$

By substituting (A2) into (A3), the Lagrange multiplier can be obtained as

(A4) $$\begin{array}{c} \begin{array}{c} \lambda^{T}=-2{\left(GG^{T}\right)}^{-1}b \end{array} \end{array}$$

The solution $e_{1}\left(t\right)$ can be obtained from (A2) by substituting (A4):

(A5) $$\begin{array}{c} \begin{array}{c} e_{1}\left(t\right)=G^{T}{\left(GG^{T}\right)}^{-1}b=G^{+}b \end{array} \end{array}$$

## Appendix B. Sensor Fault Isolation with Minimal *ℓ*_1_ -Norm Solution

In the case of the sensor faults, the response can be represented in the *s*-domain as follows:

(A6) $$\begin{array}{c} \begin{array}{c} \left(I+\alpha_{s}\right)Y_{sensor}\left(s\right)+f_{s}=G\left(s\right)U\left(s\right) \end{array} \end{array}$$

From the above equation, residual can be generated as follows:

(A7) $$\begin{array}{c} \begin{array}{c} R\left(s\right)=Y_{sensor}\left(s\right)-Y_{model}\left(s\right)\\ \;\;\;\;=\left[G\left(s\right)U\left(s\right)\right]-\left[\alpha_{s}Y_{sensor}\left(s\right)+f_{s}\right]-\left[G\left(s\right)U\left(s\right)\right]\\ \;\;\;\;=-[\alpha_{s}Y_{sensor}\left(s\right)+f_{s}] \end{array} \end{array}$$

where $R\left(s\right)\in R^{p\times 1}$.

The representative characteristics of sensor fault is that residual is independent from the transfer function *G*(*s*) and input *U*(*s*).

The residual can be represented with actuator faults as follows:

(A8) $$\begin{array}{c} \begin{array}{c} R\left(s\right)=G\left(s\right)\left[\alpha U\left(s\right)+f_{u}\right]-\left[\alpha_{s}Y_{sensor}\left(s\right)+f_{s}\right] \end{array} \end{array}$$

For the perspective of linear programming, the term independent of the transfer function G(s) is regarded as noise. Therefore, the sensor faults will not be represented in the minimal $\ell_{1}$-norm solution although the residual is actually perturbed from the sensor faults. With these characteristics, the sensor faults and actuator faults can be distinguished by the following procedure.

1. If the residual value is nonzero due to the faults, obtain fault isolation results from the minimal $\ell_{1}$-norm solution.
2. If the fault isolation results are nonzero, we can decide that there are actuator faults in an isolated location.
3. If the fault isolation results are zero in spite of nonzero residual occurrence, then we can decide that there are sensor faults rather than actuator faults. Furthermore, for the case of sensor faults, the position of nonzero residual components are directly the locations of sensor faults. Therefore, sensor fault isolation can be achieved easily.
